# Use of Machine Learning and Infrared Spectra for Rheological Characterization *and Application to the Apricot*

**DOI:** 10.1038/s41598-019-55543-7

**Published:** 2019-12-16

**Authors:** Xavier F. Cadet, Ophélie Lo-Thong, Sylvie Bureau, Reda Dehak, Miloud Bessafi

**Affiliations:** 1 PEACCEL, Protein Engineering Accelerator, 6 square Albin Cachot, box 42, 75013 Paris, France; 2 LSE laboratory, EPITA, Paris, 94276 France; 3 University of Paris, UMR_S1134, BIGR, Inserm, F-75015 Paris, France; 4 DSIMB, UMR_S1134, BIGR, Inserm, Laboratory of Excellence GR-Ex, Faculty of Sciences and Technology, University of La Reunion, F-97715 Saint-Denis, France; 5 UMR408 SQPOV, Sécurité et Qualité des Produits d’Origine Végétale, INRA, Avignon University, F-84000 Avignon, France; 6 LE2P, Laboratory of Energy, Electronics and Processes EA 4079, Faculty of Sciences and Technology, University of La Reunion, 97444 St Denis Cedex, France

**Keywords:** Biophysics, Infrared spectroscopy

## Abstract

Fast advancement of machine learning methods and constant growth of the areas of application open up new horizons for large data management and processing. Among the various types of data available for analysis, the Fourier Transform InfraRed (FTIR) spectroscopy spectra are very challenging datasets to consider. In this study, machine learning is used to analyze and predict a rheological parameter: firmness. Various statistics have been gathered including both chemistry (such as ethylene, titrable acidity or sugars) and spectra values to visualize and analyze a dataset of 731 biological samples. Two-dimensional (2D) and three-dimensional (3D) principal component analyses (PCA) are used to evaluate their ability to discriminate for one parameter: firmness. Partial least squared regression (PLSR) modeling has been carried out to predict the rheological parameter using either sixteen physicochemical parameters or only the infrared spectra. We show that (*i)* the spectra alone allows good discrimination of the samples based on rheology, (*ii)* 3D-PCA allows comprehensive and informative visualization of the data, and (*iii)* that the rheological parameters are predicted accurately using a regression method such as PLSR; instead of using chemical parameters which are laborious to obtain, Mid-FTIR spectra gathering all physicochemical information could be used for efficient prediction of firmness. As a conclusion, rheological and chemical parameters allow good discrimination of the samples according to their firmness. However, using only the IR spectra leads to better results. A good predictive model was built for the prediction of the firmness of the fruit, and we reached a coefficient of determination R^2^ value of 0.90. This method outperforms a model based on physicochemical descriptors only. Such an approach could be very helpful to technologists and farmers.

## Introduction

Fast advancement of machine learning methods and constant growth of the areas of application open up new horizons for large data management and processing^1^. Machine learning provides better results in many fields, where the problem become hard for classical approaches, such as medicine, with the speeding up of drug development and the design of chronic disease predictive models^2,3^; green chemistry and renewable energy by providing optimized materials for CO_2_ capture^4,5^ or in the field of agri-food to contribute to smart farming and assess food quality^6–10^.

Among the various types of data available for analysis, the Fourier Transform InfraRed spectroscopy (FTIR) spectra are very interesting to consider. In fact, FTIR is a rapid technique to analyze and provide high quality spectra, that presents a wide range of applications^11^. Some of these applications include the tracking of an enzymatic reaction, as well as an enzymatic assay^12^, but also molecule quantification^13,14^, the identification of different wheat grain varieties^15^ and the molecular characterization of archeological wood^16^. Recently, the method of Attenuated Total Reflectance (ATR) combined with FTIR was applied to apricots from eight different cultivars, to simultaneously determine sugar and organic acid contents and appraise the quality of these fruits^17^. Using partial least square (PLS) models, this study allowed the prediction of some quality traits including, *inter alia*, contents of sucrose, glucose, malic acid, ethylene production rate, firmness and other rheological parameters.

In order to extend the analysis, in this study particular attention will be paid to the employment of machine learning approaches to analyze and predict the physicochemical parameters of these fruits, and particularly rheological properties which are tedious to measure. Numerous studies in the literature have demonstrated the usefulness of spectroscopy techniques (Mid-infrared: MIR, Near Infrared: NIR) to predict and/or classify the rheological parameters of cheese (texture, flavor and structure)^18^, wheat^19,20^ or sludge (viscosity, elastic and viscous moduli)^21^, and a nondestructive approach has been tested using NIR spectroscopy for apples^22^.

In this work, the objectives are to:

- visualize dataset samples (731 samples) according to the physicochemical data and the spectra; PCA will be used for visualization;
- examine the results through 2D and 3D visualization and evaluate if they could be discriminated;
- predict the rheological parameter “Firmness”;
- see to what extent chemical parameters could be replaced by Mid-FTIR spectra to predict the firmness.

Collectively, the different analyses carried out show that (*i)* the spectra alone allow good discrimination of the samples based on their rheology, (*ii)* 3D-PCA allows comprehensive and informative visualization of the data, and (*iii)* the rheological parameters are predicted accurately using a regression method such as PLSR. We show that instead of using chemical parameters which are laborious to obtain, Mid-FTIR spectra gathering all physicochemical information could be used for efficient prediction.

## Materials and Methods

### Dataset

The dataset on which this work relies has been produced by Bureau *et al*.^17^. All data including spectra, chemical and rheological properties were acquired in 2005 on a large collection of apricot fruits representative of variability in terms of color, taste, texture and ability to be stored (In total, 731 fruits were individually characterized from eight varieties at different maturity stages). Some properties were determined on intact fruits, the day of harvest, such as firmness, color and ethylene production. After these measurements, fruits were cut in pieces and frozen at −20 °C. A few days later, the spectral and biochemical measurements were performed on homogeneous fruit samples after thawing and grinding. All data were acquired on each apricot at the same time to have the best relationship between spectra and the 23 measured properties. The whole spectral collection included 731 ATR-FTIR spectra, each corresponding to a fruit. The dataset has been randomly split into two sets, the Training set representing 80% (584 spectra) and the Test set representing 20% (147 spectra). A stratified separation was made according to the targeted parameter, as predictions were only made for firmness.

For each spectrum, a total of 23 chemical and rheological properties have been characterized.

Eight apricot cultivars or hybrids, named ‘Moniqui’, ‘Goldrich’, ‘Bergeron’, ‘Iranien’, ‘Badami’, ‘Ravicille’, ‘Ravilong’ and ‘A4034’ were chosen for their contrasted fruit quality traits such as colour, taste, physiological behaviour. Fruits came from two INRA experimental orchards (Amarine (Gard) and Gotheron (Drôme), South of France) for ‘Moniqui’, ‘Goldrich’, ‘Iranien’, ‘Badami’, ‘Bergeron’ and ‘A4034’, and from a traditional private orchard (Donzère, Drôme, South of France) for ‘Ravicille’ and ‘Ravilong’.

### Preprocessing of the data

#### On the chemistry and rheology data no outliers were detected

Spectra processing: the ATR-FTIR reflectance data were transformed with Standard Normal Variate (SNV) to correct multiplicative interferences, variations in baseline shift and curvilinearity^23^.

### Performances metrics

We need to establish various metrics to evaluate the proposed solution. Here, the goal is to predict a physical property, namely firmness, given IR spectra. Since we have labels, and these labels are real continuous values, we opted for a Regression task. The metrics that are more suitable in this case are the coefficient of determination (R^2^) and the Root Mean Squared Error (RMSE).

### Statistical measures of correlation

The correlation coefficient R (Pearson) is used to assess the correlation between the rheological and chemical parameters, and to identify the wavenumbers best correlated with the targeted property.

Also, the correlation between the measured and predicted values of the firmness is appraised, quantitatively and qualitatively, through the determination of R^2^ and RMSE in cross-validation (respectively cvR^2^ and cvRMSE). These statistical metrics are calculated for all of the training sets we used and help to construct and select the best models. While, R^2^ measures concordance between the measured and the predicted fitness values and therefore reveals the predictive strength of the model, RMSE measures the error of the model to predict the fitness. R^2^ and RMSE are calculated as follows in Eqs. (1) and (2) respectively:

1 $${R}^{2}(y,\hat{y})=1-\frac{{\sum }_{i=1}^{n}\,{({y}_{i}-{\hat{y}}_{i})}^{2}}{{\sum }_{i=1}^{n}\,{({y}_{i}-\bar{y})}^{2}}$$

2 $$RMSE=\sqrt{\mathop{\sum }\limits_{i=1}^{n}\frac{{({y}_{i}-\widehat{{y}_{i}})}^{2}}{n}}$$

where, *y*_*i*_ is the measured activity of the *i*^th^ sequence, *ŷ*_*i*_ is the predicted firmness of the *i*^th^ sequence, *ȳ* is the average of the measured activities and *n* the number of sequences in the test set.

Also, cvRMSE makes it possible to identify the best predictive models among all the models designed and represents the extent to which the predictions vary when different training sets are used.

### Modeling and prediction schema

Physicochemical traits can be considered as fitness traits including firmness, skin colour, ethylene production, soluble solids content and titratable acidity…

We use the FTIR spectra and the fitness (23 traits are used in this work) as inputs for the learning process of our model. Then, the aim of this learning is to set up a statistical model that links the fitness to the FITR spectra. In fact, the model uses the initial dataset (training set: spectra and fitness traits) to model the fitness of FTIR spectra. Using the training set, a PLS regression is performed.

To perform the PLS regression, linear combinations of the original variables (i.e. input datasets) are calculated to constitute the latent components, which explain the maximum variance observed in the spectra. Also, the number of latent components to be considered for the PLS regression is based on the number of components that yield the least cvRMSE and thus present the best predictive power.

Then, the statistical model, obtained by the PLS regression method on the training dataset, is used to predict the firmness of the test dataset. Two methods of cross-validation are performed on the dataset: Leave-One-Out Cross-Validation (LOOCV) and 80–20 partitioning (80% training set and 20% validation set). And the efficiency of the predictions (test set) is evaluated using the previously discussed statistical parameters R^2^ and RMSE.

For proper model validation we withheld a test set. This test set has never been used during the model training. In other words, the test set was put an *inner sanctum* and nobody knew what was the spectra inside and/or the values of the parameter associated with the spectra and/or with the chemical properties. After model training, the final model was used to predict the latent variables of this test set. This approach is the best machine learning practice to evaluate the capacity of generalization of the trained model.

Performances metrics, Statistical measures of correlation and Modeling and prediction schema have been fully described in our preceding paper^24^.

All the scripts have been written in Python.

## Results and Discussion

Our working hypothesis was the following: the firmness is the sum of different parameters depending on the level of chemical compounds in the fruit: fibers, sugars, acids, water… The quantification of these parameters would be very helpful, but their chemical or physical determination could be laborious, costly and sometimes difficult to perform. Such measurements usually require an experienced chemist, a lot of time and chemical consumables. On the other hand, Infra-Red techniques such as ATR will provide a spectrum which takes into account all the physicochemical information. This is very simple to handle: a scan can be performed in a few seconds.

Our hypothesis is that a spectrum is sufficient for the determination of a rheological parameter such as firmness.

### Data exploration

#### Distribution analysis

**Figure 1:**
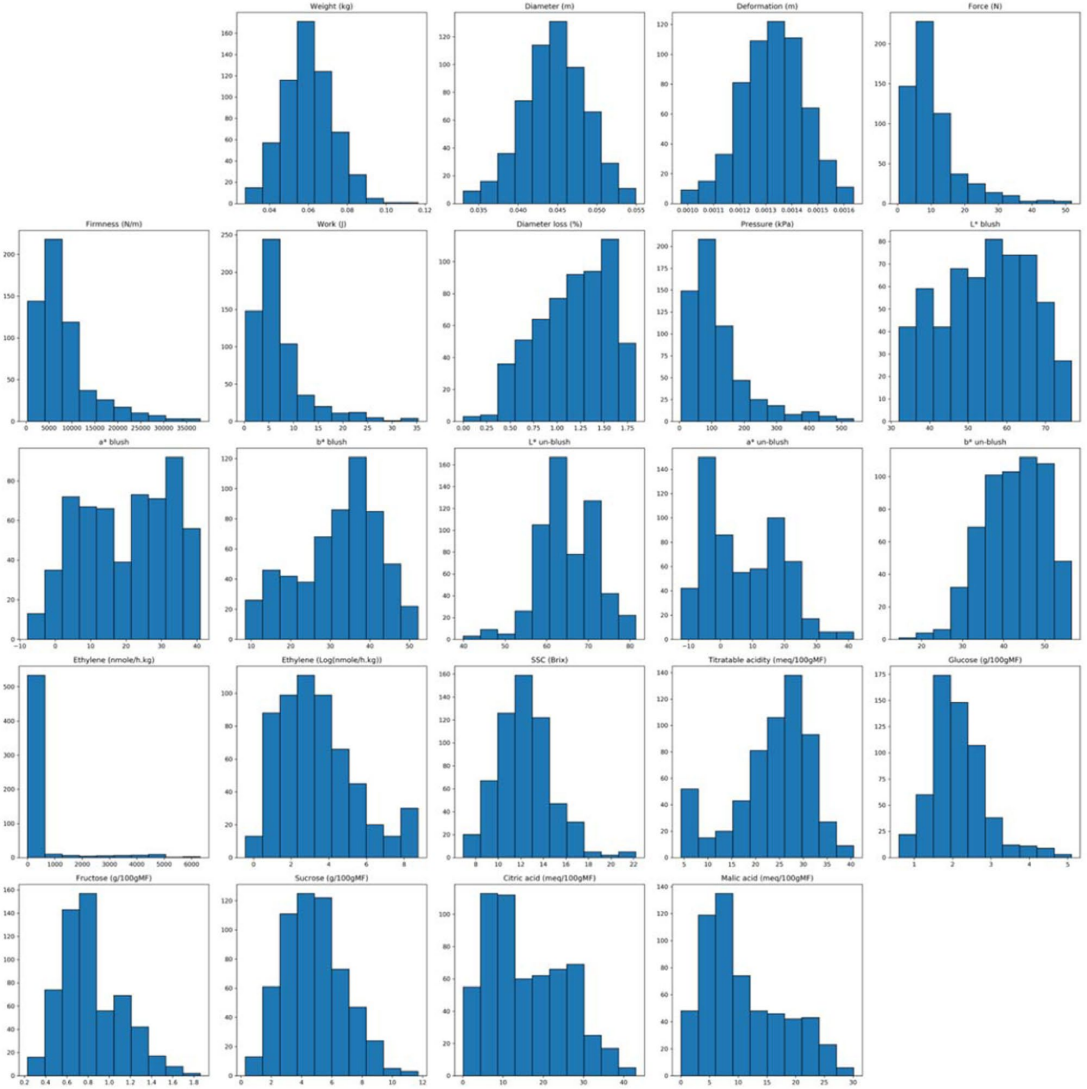
Chemical and rheological properties distributions. 23 properties are displayed. From the top down and from left to right the parameters are the following: weight (kg), diameter (m), deformation (m), force (N), firmness (N/m), work (J), diameter loss (%), pressure (kPa), L*blush, a*blush, b*blush, L*un-blush, a*un-blush, b*un-blush, ethylene (nmole/h.kg), ethylene (Log(nmole/h.kg)), SSC (Brix), titrable acidity (meq/100gFW), glucose (g/100gFW), fructose (g/100gFW), sucrose (g/100gFW), citric acid (meq/100gFW) and malic acid (meq/100gFW). SSC: soluble solids content, FW: fresh weight.

Chemical and rheological properties distributions of the 584 ATR-FTIR spectra are presented in Fig. 1.

Different types of probability distribution appear. As an example: normal distribution (weight), beta distribution (b* un-blush) or gamma distribution (sucrose). It should be pointed out that the property “Firmness” follows a log-normal which takes on values whose logarithm is normally distributed. The exponentiation of a normally-distributed value is log-normally distributed. Since PLS performs well on normal distribution, preprocessing using logarithm function can be applied on the firmness data and regression methods such a PLSR will be explored for the modeling.

The distribution gives an idea of distribution parameters but does not provide any information about the correlation between parameters. Thus, various statistics have been gathered over both chemistry and spectra values and are exhibited in the following sections.

#### Correlations between the properties

**Figure 2:**
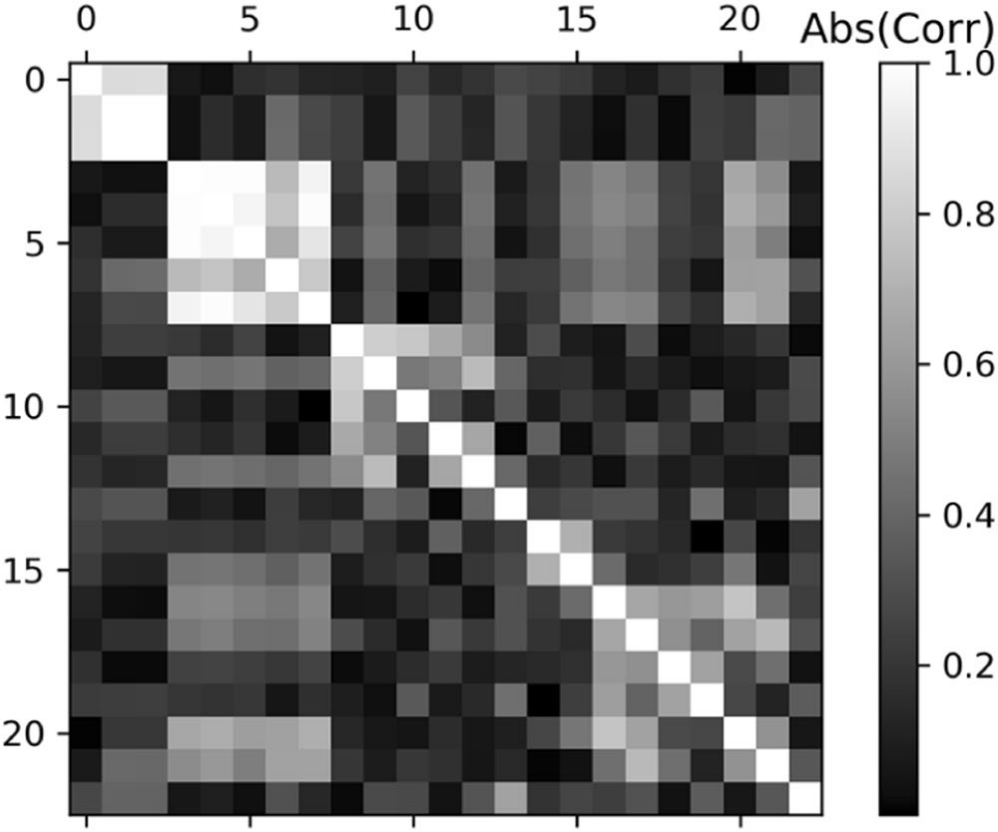
Chemical and rheological Correlations (Absolute values). The 23 properties shown in Fig. 1 are numbered 1 to 23.

Correlations have been observed (Fig. 2), first over the entire train set. The diagonal related variances do not give any relevant information.

The strong correlations around the 5^th^ component can easily be explained, as some are combinations of the others. Indeed, the firmness parameter can be expressed as the Force (N) over the Deformation (m). The parameters of firmness are obtained using a 3% deformation test of equatorial height of the fruit with a multi-purpose texture analyzer (Pénélaup, Serisud, Montpellier, France). Otherwise, interesting correlations are shown. The quantitative values of the correlation coefficient R are presented in Table 1 for firmness. Positive and negative correlations are observed. Some can be easily understood: for example, firmness has a negative correlation with the sucrose content. We can assume that the more mature the fruit, the less firm it will be. Other correlations have to be explored.

**Table 1:** Firmness correlations with the other fitness traits.

Fitness	Firmness (N/m)
Firmness (N/m)	1.00
Force (N)	0.99
Pressure (kPa)	0.99
Work (J)	0.96
Citric acid (meq/100gFW)	0.60
Titratable acidity (meq/100gFW)	0.50
L* blush	0.16
L* un-blush	0.13
b* blush	0.06
Weight (kg)	−0.03
Malic acid (meq/100gFW)	−0.10
b* un-blush	−0.11
Deformation (m)	−0.16
Diameter (m)	−0.16
Fructose (g/100gFW)	−0.19
Ethylene (nmole/h.kg)	−0.21
Glucose (g/100gFW)	−0.26
a* blush	−0.43
a* un-blush	−0.46
Ethylene (Log(nmole/h.kg))	−0.46
SSC (Brix)	−0.54
Sucrose (g/100gFW)	−0.69
Diameter loss (%)	−0.77

**Figure 3:**
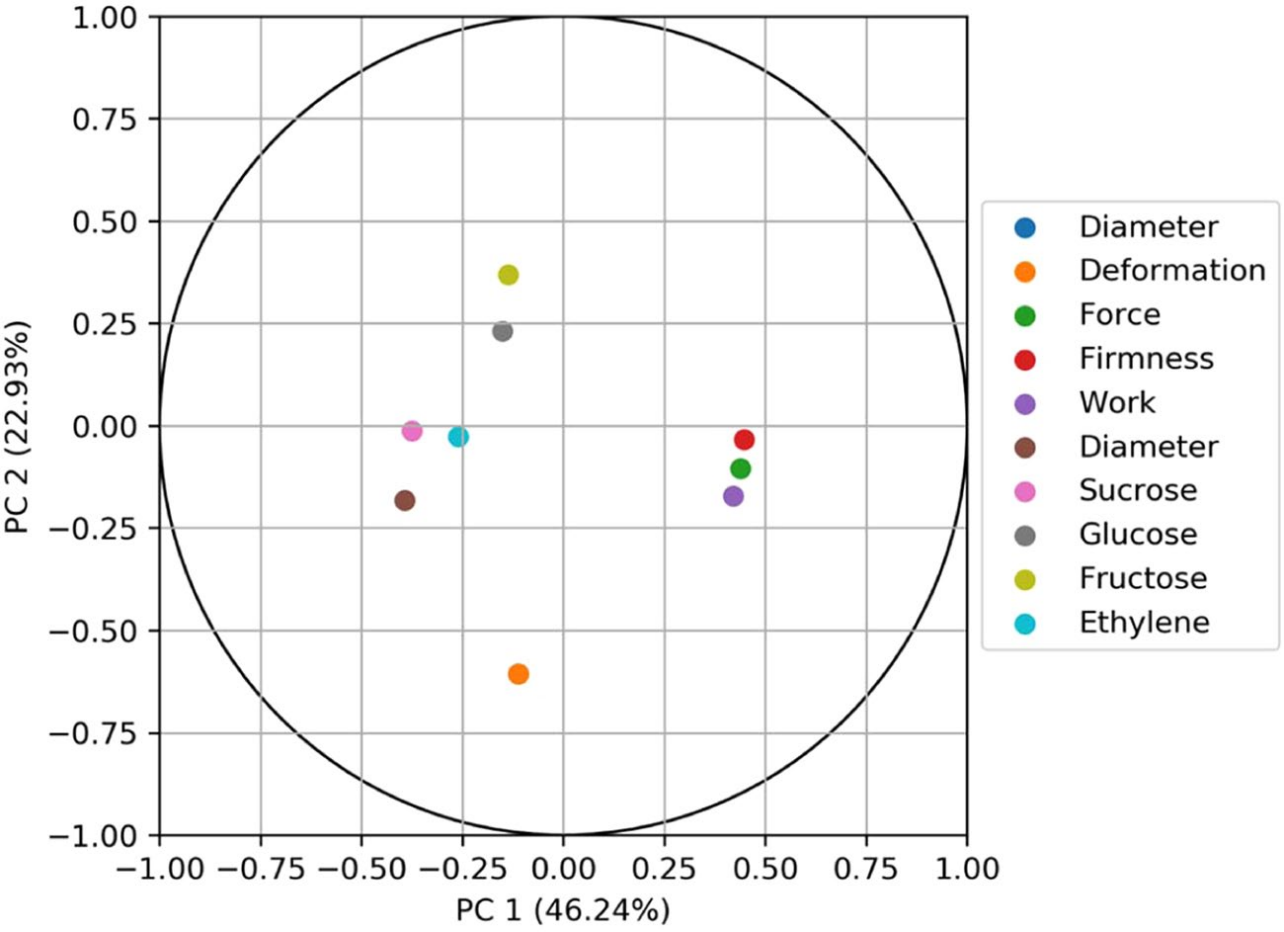
Correlation plots with first principal component (PC1. 46.24%) and second principal component (PC2. 22.93%).

Principal Component Analysis (PCA) is useful to observe how the physical quantities are organized in a new space and to observe potential similarities between them. The correlation plot is presented in Fig. 3. It shows that the principal component 1 (PC1) is positively correlated with firmness and negatively to sucrose or ethylene, which are markers for ripeness. Firmness is close to force. The second principal component (PC2) is mainly negatively correlated to deformation and positively correlated to fructose, and neutral regarding firmness.

**Figure 4:**
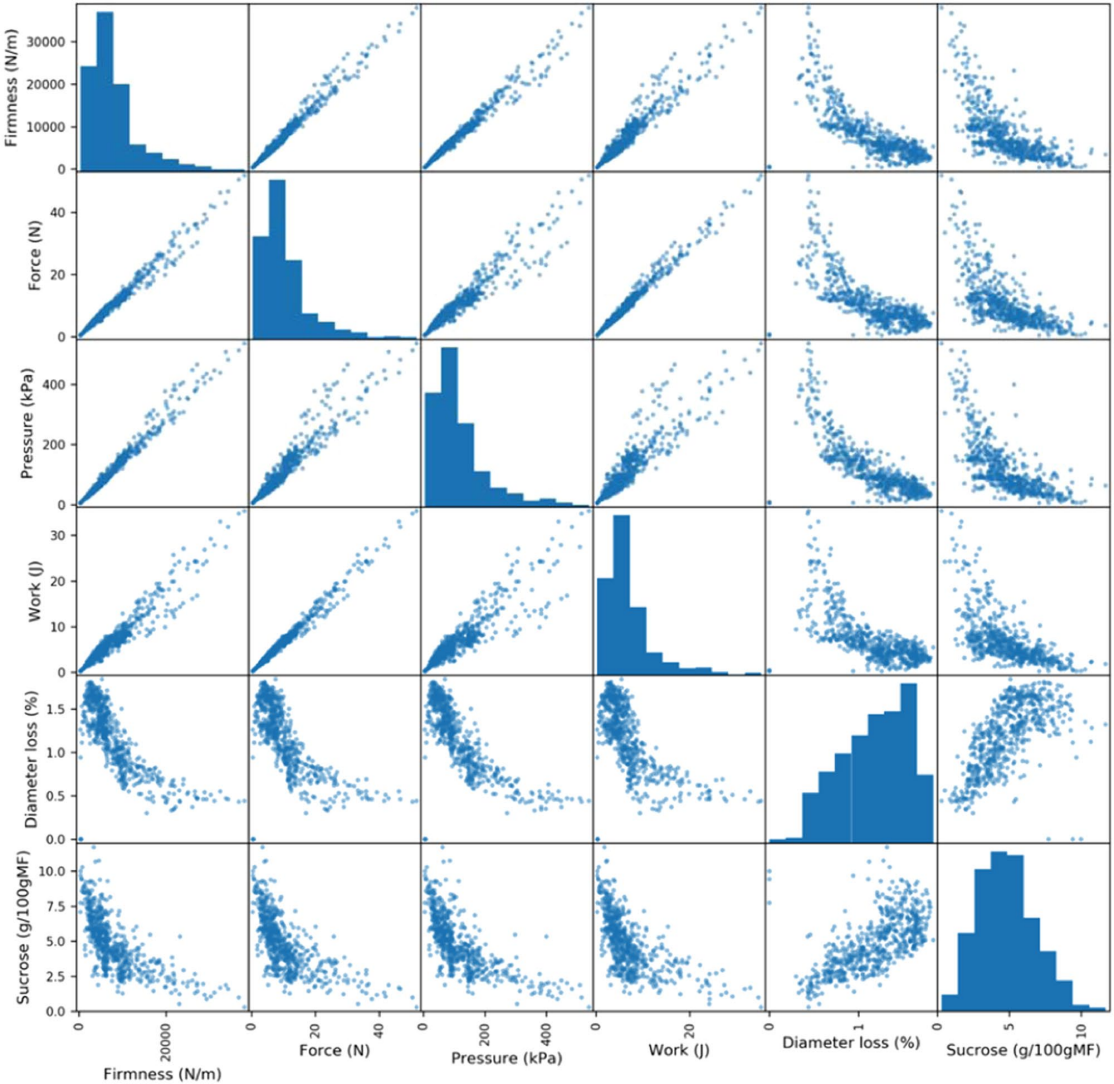
Firmness top 5 absolute correlation scatter matrix.

Figure 4 presents Firmness: top five correlations (positive or negative) in relation to other fitness.

For a set of data variables of dimensions x_1_… x_k_, the scatter matrix exhibits all the pairwise scatter plots of the variables on a single view with multiple scatterplots in a matrix format. For k variables, the scatterplot matrix contains k rows and k columns. A plot located at the intersection of i^th^ row and j^th^ column is a plot of variables x_i_ versus x_j_. Paired combinations of qualitative and quantitative variables can be examined globally. In our case, the diagonal has been replaced by the histogram associated with the value instead of displaying a non-informative diagonal. Figure 4 shows linear (pressure, work…), non-linear (exponential pattern with sucrose or diameter loss), positive or negative correlations. The shape of the plots gives an idea of the kind of preprocessing or regression model to perform for the next steps. As an example, Figure 4 shows (*i)* a linear relation between pressure and firmness that could be easily modeled using a linear regression model or (*ii)* a non-linear relation between sucrose and firmness suggesting, for example, that a preprocessing step applied to sucrose data, such as Logarithm function, in order to linearize the sucrose data before applying a regression model, should improve the results. Such information is interesting in the sense that if we have the measurements for the pressure, then it will be possible to predict firmness, but more interestingly, that the results obtained with spectra and firmness (see section*Models and Predictions*), would probably be similar for the couple spectra and pressure.

**Figure 5:**
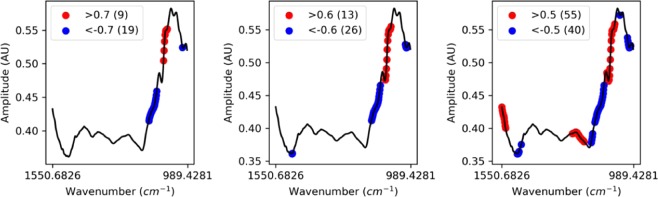
Firmness and mean SNV spectra correlations.

Figure 5 is composed of three figures; the red points are wavenumbers associated with positive correlations above a given threshold, and blue dots are associated with negative correlations below the opposite threshold. The number in parentheses is the number of wavenumbers associated with such correlations. From left to right (threshold: 0.7, 0.6 and 0.5). The black line corresponds to the mean of the different spectra.

Table 2 pinpoints wavenumbers that are the most closely correlated to firmness.

**Table 2:** Wavenumbers correlated to firmness, with a coefficient of correlation superior to the absolute value 0.5. (+) for positive correlation and (−) for negative correlation.

wavenumber (cm^−1^)	Sign of correlation	Molecules	wavenumber (cm^−1)^	Molecules	Sign of correlation
1550	+	acids	1010	sucrose	−
1548	+	acids	1008	sucrose	−
1546	+	acids	1006	sucrose	−
1544	+	acids	1004	sucrose	−
1542	+	acids	1002	sucrose	−
1541	+	acids	1001	sucrose	−
1539	+	acids	999	sucrose	−
1020	−	glucose	997	sucrose	−
1018	−	glucose	995	sucrose	−
1016	−	glucose	993	sucrose	−
1014	−	sucrose	991	sucrose	−
1012	−	sucrose	989	sucrose	−

Wavenumbers positively correlated to firmness (red points in Fig. 5) which range between 1550 and 1539 cm^−1^ correspond mainly to organic acids^17^. Among the ones negatively correlated to firmness (blue points in Fig. 5), those which range between 1020 to 1016 cm^−1^ correspond mainly to glucose absorption and between 989 to 1014 cm^−1^ to sucrose^25^. These observations are consistent with the results shown in Table 1.

Further analysis of the spectra is described in the *Visualization* section.

### Data preprocessing

As observed in the correlation analysis (Fig. 1), and given further insight on the different properties gathered, the parameter “Ethylene” was replaced by Log (Ethylene). However, these chemicals are only used for visualization purposes, as the whole point is to be able to obtain proper prediction of such variables given IR spectra collected using any device.

**Figure 6:**
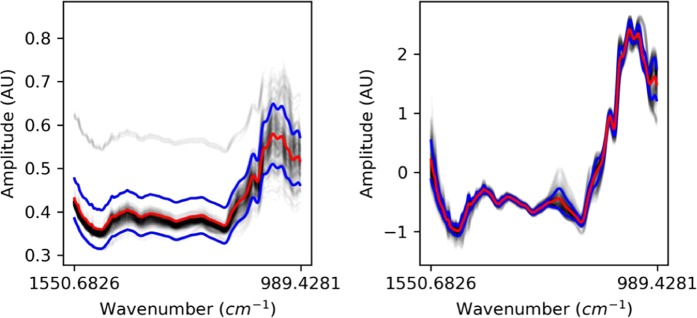
(left) Original train Spectra (right) SNV Spectra.

In Fig. 6, black transparent lines represent the different spectra, red lines represent the means of the spectra, and the blue lines represent the standard deviation of the spectra. The left-hand side plot represents the spectra from the dataset before any further modification, the right-hand plot represents the spectra after individual SNV. SNV spectra (right) are used for the next steps.

### Visualization

PCA was performed with either the Fitness traits or the spectra. Factorial maps with two or three principal components are exhibited so as to explore how the data are distributed.

#### 2D-PCA

**Figure 7:**
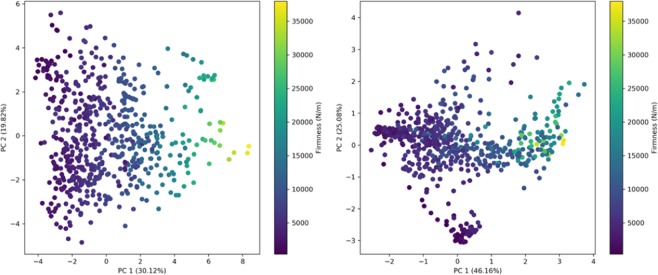
2D-PCA Chemistry and Rheology (left) and Spectra (right) with respect to Firmness.

A PCA was run on the dataset so as to have a global visualization of the spectra. Figure 7 (left) shows the PCA using the chemical and rheological parameters. The gradient of colour indicates the firmness. We can see a discrimination of the samples according to firmness. The principal component 1 gathers 30.12% of the information, the second 19.82%. Figure 7 (right) presents an image with more distinct representation but with the spectra. PC1 has a percentage of inertia of 46.16%, PC2 25.08%. In this case, fewer axes gather more of the information embedded in the samples. When the data points are plotted according to firmness, a gradient is revealed. This suggests that spectra could be a good descriptor for predicting firmness.

These 2D plots could hide overlaps and could be misleading. Hence, we decided to plot with three axes.

#### 3D-PCA

**Figure 8:**
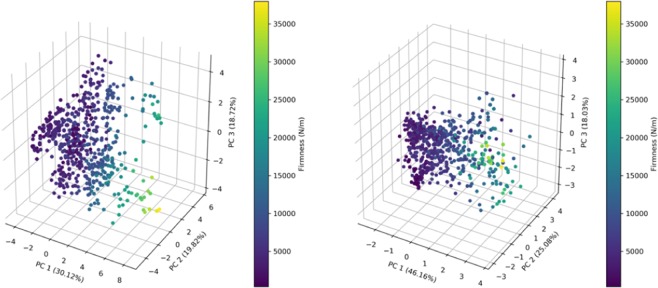
3D-PCA Chemistry and Rheology (left) and Spectra (right) with respect to Firmness.

3D visualization in Fig. 8 (left) based on chemistry and rheology uncovers overlaps and makes the distribution of the spectra more comprehensible. It has been shown^17^ that such distribution is mainly guided by the origin of the fruit. Similar results are obtained in Fig. 8 (right) where spectra are used, confirming that a good distribution of the Firmness is obtained. Discrimination among firmness values is more distinct. Once again, the gradient spectra observed suggest that spectra could be a good descriptor to predict firmness.

**Figure 9:**
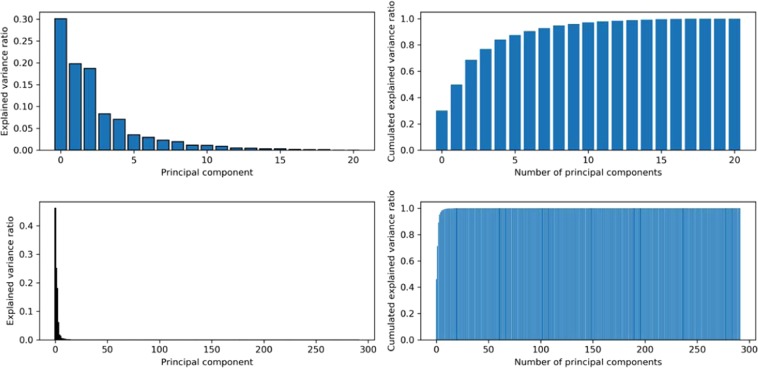
Explained variance per principal component and cumulated explained variance: (upper) using the physicochemical parameters (Firmness and ethylene are excluded), (lower) using the spectra.

Figure 9 shows a comparison of the percentage of inertia (left) associated with each component from a PCA and the cumulated components (right side) when (*i)* all the physicochemical parameters are used except firmness and ethylene (as Log (ethylene) is kept), and (*ii)* when the spectra are used. The spectra appear to be very informative, as a few components provide almost the full information. On the contrary, each physicochemical parameter has to be taken into account to explain all the information contained in the samples.

In order to explore how efficient a prediction of the firmness based on spectra would be, we decided to carry out regression models.

As described earlier, the dataset has been randomly divided into a train set and a test set with 80%/20% split.

### Models and predictions

As suggested in the section “*Distribution analysis”*, Log(Firmness) has been used for the modeling.

#### Type of learning

As stated before, when defining the metrics, given the fact that we have labels which are continuous, the task is a supervised learning task, and more specifically a Regression task. Performances have to be established based on the metrics discussed in the *Metric* section, R^2^ and RMSE.

#### Cross-validation

In order to calibrate the models, Cross-Validation has been performed with a Leave-One-Out approach. The fine-tuning step has been carried out via comparison of the metrics through Leave-One-Out, to determine the best parameters for the model.

#### Model selection

A Partial Least Squared Regression (PLSR) is used.

#### Fine-tuning

Leave-One-Out Cross validation comparison was used to determine the best number of components, the number of components that minimizes the RMSE. A threshold of 40 components at most was set.

**Figure 10:**
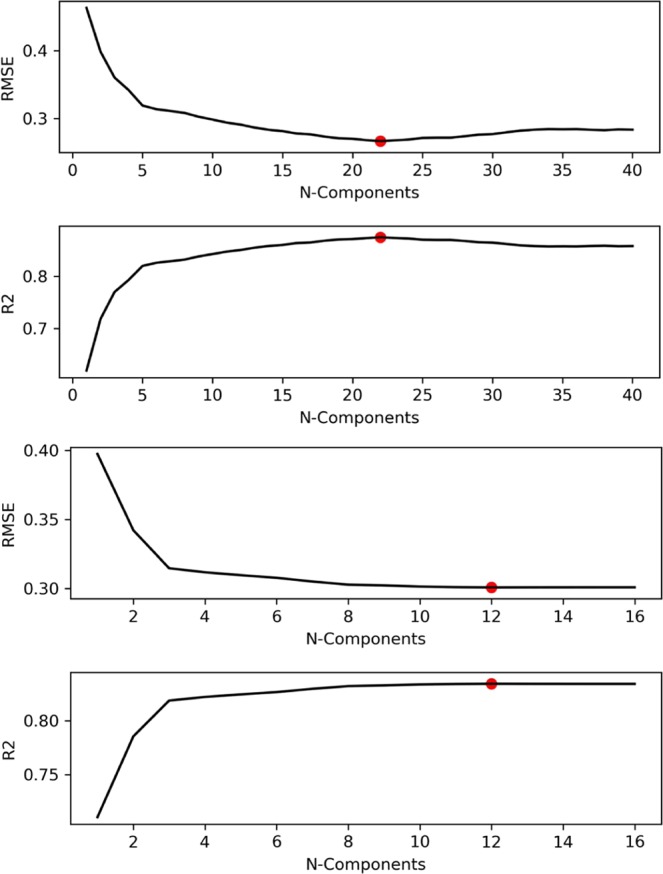
(**a,b**) RMSE (upper plot) and R^2^ (lower plot) given N-Components during PLSR. (**a**) using spectra: two upper plots and, (**b**) two lower plots, using physicochemical parameters (firmness and rheological parametersare excluded).

Figure 10 shows that the best number of components to choose for the PLSR model is 22 (among 291) with spectra (upper) and 12 (out of 16) with the physicochemical parameters: this number of components leads to the lowest cvRMSE.

**Figure 11:**
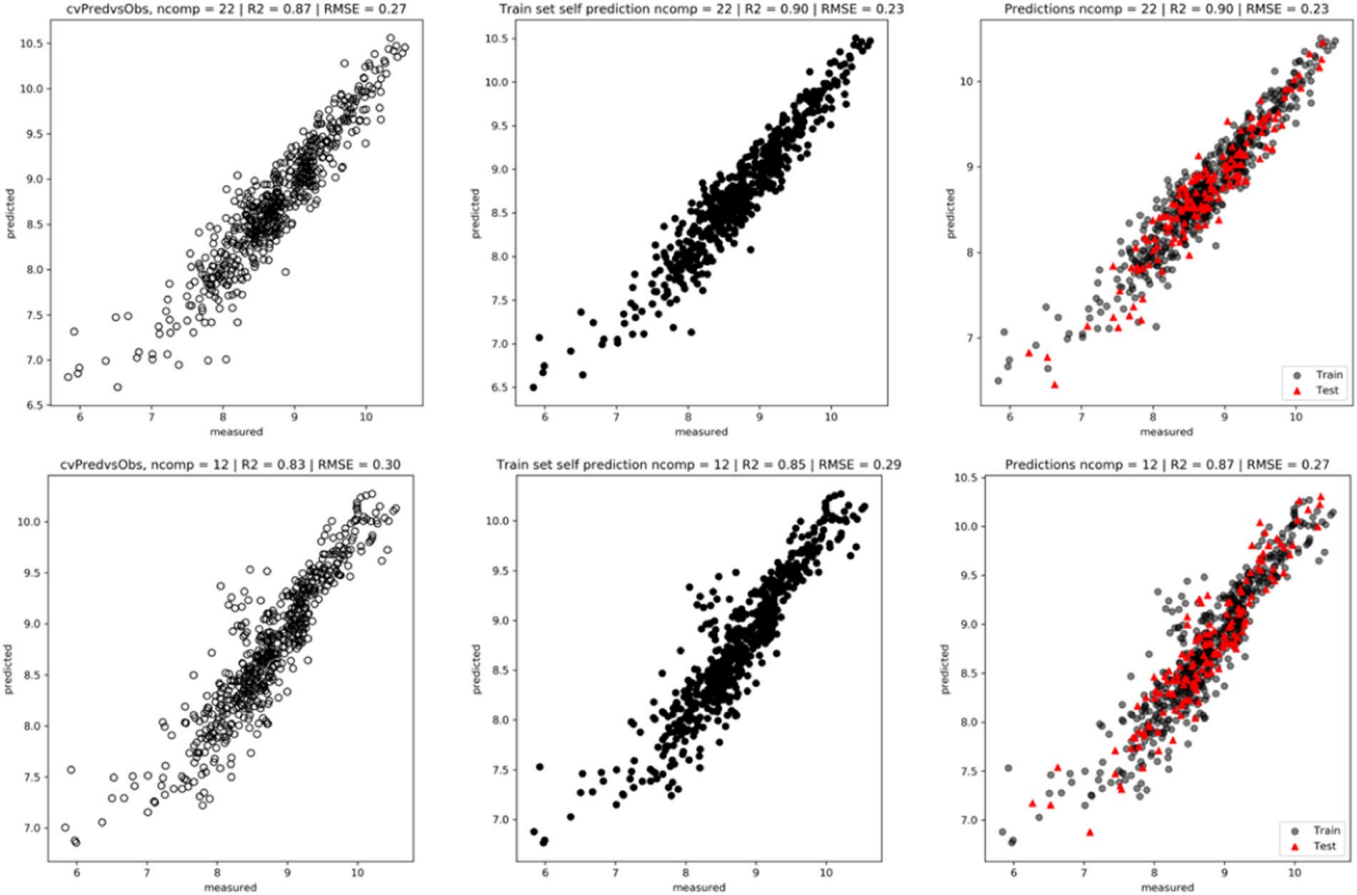
(**a,b**) Predictions. LOOCV procedure applied on the train set (left). Prediction of the train set using the model with 22 components (center). Prediction of the test set (right) in red. (**a**) With spectra (upper plots) and (**b**) physicochemical parameters (lower plots) (firmness and rheological parameters are excluded).

Figure 11 (left) shows the plots obtained after a LOOCV procedure: cvR^2^: 0.87 and cvRMSE: 0.27 with spectra (upper) and 0.83/0.30 with physicochemical parameters (lower). The model using spectra gives better predictions. Figure 11 (center) shows a prediction of the Train set using the best model obtained with the LOOCV procedure. As expected, the predictions are good. Figure 11 (right) shows the prediction of the Test set (20%) using the Train set (80%): R^2^: 0.90 and RMSE: 0.23 versus 0.87/0.27 confirm that a model built with the spectra outperforms the one built with physicochemical parameters for the prediction of firmness. This is not surprising since a FTIR spectrum contains at least the information of the chemical compounds used as descriptors.

## Conclusion

In this study, we show that:

1. Rheological and chemical parameters allow good discrimination of the samples of apricots according to their firmness.
2. However, using only the IR spectra leads to better results. Good discrimination is obtained in both cases between the samples according to firmness.
3. A predictive model based on IR spectra demonstrates its ability to efficiently predict firmness. Wavenumbers positively or negatively correlated to the Firmness were pointed out.

As a conclusion: a good predictive model was built for the prediction of fruit firmness, as we obtained an R^2^ value of 0.90. This model outperforms a model based on physicochemical descriptors only. This approach could be very helpful to technologists or farmers. It could be extended to other fruits and vegetables. It could open exciting perspectives for fast quantitative rheological characterization of other kinds of material in various industries: ceramic, plastic, composites, polymers of small chemical or biological compounds…

## Data Availability

The datasets used in this study are fully described in the manuscript.
